# Supplementary material for: Exploring health inequalities by language proficiency; A routine health records study set in England
Source: PLoS One. 2026 Aug 19;21(8):e0342624. doi: 10.1371/journal.pone.0342624 (PMC13489480; doi:10.1371/journal.pone.0342624)
Supplement: Supplementary file 1 [file pone.0342624.s001.docx]

**Supplementary Material**

**Table S1** Recorded first languages

| First Language^1^ | NP  N = 9,403 | NP-Previous  N = 12,836 | P-Other  N = 91,999 |
| --- | --- | --- | --- |
| Other | 2,492 (27%) | 2,583 (20%) | 27,048 (29%) |
| Gujarati | 1,219 (13%) | 1,894 (15%) | 19,300 (21%) |
| Arabic | 2,065 (22%) | 1,665 (13%) | 8,708 (9.5%) |
| Romanian | 587 (6.2%) | 918 (7.2%) | 7,578 (8.2%) |
| Portuguese | 872 (9.3%) | 978 (7.6%) | 6,134 (6.7%) |
| Somali | 493 (5.2%) | 858 (6.7%) | 4,495 (4.9%) |
| Polish | 311 (3.3%) | 625 (4.9%) | 4,292 (4.7%) |
| Urdu | 198 (2.1%) | 385 (3.0%) | 4,301 (4.7%) |
| Hindi | 133 (1.4%) | 195 (1.5%) | 4,230 (4.6%) |
| Tamil | 179 (1.9%) | 1,015 (7.9%) | 3,300 (3.6%) |
| Italian | 54 (0.6%) | 166 (1.3%) | 2,613 (2.8%) |
| Language not recorded | 800 (8.5%) | 1,554 (12%) | 0 (0%) |
| ^1^n (%) |  |  |  |

P-Other = Proficient in English, but English is not their first language; NP = Not proficient in English; NP-Previous = Not proficient in English, captured previously in GP record
