# Supplementary material for: Exploring health inequalities by language proficiency; A routine health records study set in England
Source: PLoS One. 2026 Aug 19;21(8):e0342624. doi: 10.1371/journal.pone.0342624 (PMC13489480; doi:10.1371/journal.pone.0342624)
Supplement: Supplementary file 2 [file pone.0342624.s002.docx]

**Supplementary Material**

**Table S2** Association between healthcare utilisation and language proficiency among adults in Brent, adjusted for ethnicity.

| Characteristic | GP Encounters | Hospital admissions | |
| --- | --- | --- | --- |
|  |  | Any | Multiple |
|  | Beta (95% CI)^1^ | OR (95% CI) ^1^ | OR (95% CI) ^1^ |
| **Age** | 0.00 (0.00, 0.01)*** | 1.02 (1.02, 1.02)*** | 1.03 (1.02, 1.03)*** |
| **Sex** |  |  |  |
| Female | - | - | - |
| Male | -0.19 (-0.19, -0.19)*** | 0.92 (0.89, 0.95)*** | 1.07 (1.00, 1.13)* |
| **IMD decile** |  |  |  |
| 1 (most deprived) | - | - | - |
| 2 | -0.01 (-0.01, 0.00)* | 0.91 (0.85, 0.98)** | 0.90 (0.80, 1.03) |
| 3 | 0.01 (0.00, 0.01) | 0.84 (0.78, 0.90)*** | 0.76 (0.67, 0.87)*** |
| 4 | 0.01 (0.00, 0.01)* | 0.87 (0.82, 0.94)*** | 0.79 (0.69, 0.89)*** |
| 5 | -0.01 (-0.02, -0.01)*** | 0.81 (0.75, 0.87)*** | 0.75 (0.66, 0.85)*** |
| 6 | -0.03 (-0.03, -0.02)*** | 0.77 (0.71, 0.84)*** | 0.64 (0.55, 0.74)*** |
| 7 | 0.00 (-0.01, 0.00) | 0.78 (0.70, 0.87)*** | 0.66 (0.54, 0.80)*** |
| 8 | 0.00 (-0.01, 0.01) | 0.92 (0.83, 1.03) | 0.79 (0.64, 0.96)* |
| **Number of LTCs** |  |  |  |
| 0 | - | - | - |
| 1 | 0.31 (0.31, 0.31)*** | 1.98 (1.88, 2.08)*** | 2.56 (2.30, 2.86)*** |
| 2+ | 0.50 (0.49, 0.50)*** | 3.91 (3.74, 4.09)*** | 5.94 (5.40, 6.53)*** |
| **Ethnicity** |  |  |  |
| Indian | - | - | - |
| African | 0.01 (0.01, 0.02)*** | 1.09 (1.02, 1.18)* | 1.01 (0.87, 1.16) |
| Bangladeshi | 0.09 (0.07, 0.11)*** | 1.08 (0.84, 1.36) | 1.00 (0.61, 1.54) |
| British | -0.01 (-0.01, 0.00)* | 1.14 (1.07, 1.22)*** | 1.17 (1.04, 1.31)** |
| Caribbean | 0.02 (0.01, 0.02)*** | 1.17 (1.09, 1.26)*** | 1.06 (0.93, 1.22) |
| Chinese | -0.08 (-0.09, -0.07)*** | 0.61 (0.49, 0.75)*** | 0.63 (0.39, 0.95)* |
| Irish | -0.04 (-0.05, -0.04)*** | 1.31 (1.19, 1.44)*** | 1.33 (1.13, 1.56)*** |
| Other Asian | 0.00 (0.00, 0.01) | 1.07 (1.00, 1.14) | 0.97 (0.85, 1.10) |
| Other Black | 0.03 (0.02, 0.03)*** | 1.25 (1.14, 1.37)*** | 1.21 (1.02, 1.43)* |
| Other Ethnic | 0.01 (0.00, 0.01)* | 1.15 (1.07, 1.23)*** | 1.11 (0.97, 1.27) |
| Other Mixed | -0.01 (-0.02, 0.00) | 1.04 (0.90, 1.19) | 0.88 (0.65, 1.17) |
| Other White | -0.09 (-0.09, -0.09)*** | 0.94 (0.89, 1.00)* | 0.96 (0.86, 1.07) |
| Pakistani | 0.06 (0.05, 0.06)*** | 1.31 (1.21, 1.43)*** | 1.22 (1.04, 1.43)* |
| Unknown | -0.12 (-0.12, -0.11)*** | 1.04 (0.95, 1.13) | 1.25 (1.05, 1.47)* |
| White And Asian | -0.02 (-0.04, 0.00)* | 0.63 (0.46, 0.85)** | 0.58 (0.29, 1.03) |
| White And Black African | 0.00 (-0.01, 0.02) | 1.21 (1.00, 1.45)* | 1.03 (0.70, 1.45) |
| White And Black Caribbean | 0.01 (0.00, 0.03) | 1.16 (0.98, 1.37) | 1.34 (1.01, 1.75)* |
| **English Proficiency** |  |  |  |
| P-English | - | - | - |
| P-Unknown | -0.09 (-0.09, -0.08)*** | 0.71 (0.68, 0.74)*** | 0.62 (0.57, 0.68)*** |
| P-Other | -0.02 (-0.02, -0.01)*** | 0.89 (0.85, 0.93)*** | 0.76 (0.70, 0.83)*** |
| NP-Prev | -0.02 (-0.03, -0.02)*** | 0.99 (0.91, 1.07) | 0.84 (0.72, 0.97)* |
| NP | 0.13 (0.12, 0.14)*** | 1.37 (1.26, 1.49)*** | 1.31 (1.12, 1.52)*** |
| NP-English | 0.07 (0.05 0.09)*** | 1.38 (1.15, 1.65)*** | 1.67 (1.24, 2.20)*** |
| ^1^*p<0.05; **p<0.01; ***p<0.001  Abbreviations: CI = Confidence Interval; OR = Odds Ratio | | | |

**Table S2 continued** Association between healthcare utilisation and language proficiency among adults in Brent, adjusted for ethnicity.

| Characteristic | ED attendances | | Avoidable ED attendances | |
| --- | --- | --- | --- | --- |
|  | Any | Multiple | Any | Multiple |
|  | OR (95% CI) | OR (95% CI) | OR (95% CI) | OR (95% CI) |
| **Age** | 1.00 (1.00, 1.00)*** | 1.00 (1.00, 1.00) | 1.00 (1.00, 1.00)*** | 1.00 (1.00, 1.00) |
| **Sex** |  |  |  |  |
| Female | - | - | - | - |
| Male | 0.81 (0.79, 0.82)*** | 0.82 (0.80, 0.84)*** | 0.81 (0.79, 0.82)*** | 0.82 (0.80, 0.84)*** |
| **IMD decile** |  |  |  |  |
| 1 (most deprived) | - | - | - | - |
| 2 | 0.87 (0.84, 0.91)*** | 0.84 (0.80, 0.89)*** | 0.87 (0.84, 0.91)*** | 0.84 (0.80, 0.89)*** |
| 3 | 0.80 (0.77, 0.83)*** | 0.78 (0.74, 0.83)*** | 0.80 (0.77, 0.83)*** | 0.78 (0.74, 0.83)*** |
| 4 | 0.74 (0.72, 0.77)*** | 0.70 (0.67, 0.74)*** | 0.74 (0.72, 0.77)*** | 0.70 (0.67, 0.74)*** |
| 5 | 0.73 (0.71, 0.76)*** | 0.67 (0.63, 0.70)*** | 0.73 (0.71, 0.76)*** | 0.67 (0.63, 0.70)*** |
| 6 | 0.73 (0.70, 0.76)*** | 0.65 (0.61, 0.69)*** | 0.73 (0.70, 0.76)*** | 0.65 (0.61, 0.69)*** |
| 7 | 0.72 (0.68, 0.77)*** | 0.67 (0.61, 0.72)*** | 0.72 (0.68, 0.77)*** | 0.67 (0.61, 0.72)*** |
| 8 | 0.72 (0.67, 0.77)*** | 0.63 (0.57, 0.70)*** | 0.72 (0.67, 0.77)*** | 0.63 (0.57, 0.70)*** |
| **Number of LTCs** |  |  |  |  |
| 0 | - | - | - | - |
| 1 | 1.78 (1.73, 1.82)*** | 1.89 (1.82, 1.96)*** | 1.78 (1.73, 1.82)*** | 1.89 (1.82, 1.96)*** |
| 2+ | 2.67 (2.61, 2.74)*** | 3.15 (3.05, 3.26)*** | 2.67 (2.61, 2.74)*** | 3.15 (3.05, 3.26)*** |
| **Ethnicity** |  |  |  |  |
| Indian | - | - | - | - |
| African | 1.34 (1.29, 1.40)*** | 1.43 (1.35, 1.51)*** | 1.34 (1.29, 1.40)*** | 1.43 (1.35, 1.51)*** |
| Bangladeshi | 1.34 (1.18, 1.53)*** | 1.40 (1.16, 1.67)*** | 1.34 (1.18, 1.53)*** | 1.40 (1.16, 1.67)*** |
| British | 1.16 (1.12, 1.20)*** | 1.18 (1.12, 1.25)*** | 1.16 (1.12, 1.20)*** | 1.18 (1.12, 1.25)*** |
| Caribbean | 1.38 (1.32, 1.44)*** | 1.48 (1.39, 1.58)*** | 1.38 (1.32, 1.44)*** | 1.48 (1.39, 1.58)*** |
| Chinese | 0.58 (0.53, 0.64)*** | 0.48 (0.40, 0.56)*** | 0.58 (0.53, 0.64)*** | 0.48 (0.40, 0.56)*** |
| Irish | 1.25 (1.18, 1.33)*** | 1.47 (1.35, 1.60)*** | 1.25 (1.18, 1.33)*** | 1.47 (1.35, 1.60)*** |
| Other Asian | 1.21 (1.17, 1.26)*** | 1.30 (1.23, 1.37)*** | 1.21 (1.17, 1.26)*** | 1.30 (1.23, 1.37)*** |
| Other Black | 1.45 (1.38, 1.53)*** | 1.52 (1.42, 1.63)*** | 1.45 (1.38, 1.53)*** | 1.52 (1.42, 1.63)*** |
| Other Ethnic | 1.27 (1.23, 1.32)*** | 1.36 (1.29, 1.44)*** | 1.27 (1.23, 1.32)*** | 1.36 (1.29, 1.44)*** |
| Other Mixed | 1.12 (1.04, 1.21)** | 1.26 (1.13, 1.39)*** | 1.12 (1.04, 1.21)** | 1.26 (1.13, 1.39)*** |
| Other White | 0.97 (0.94, 1.00)* | 1.05 (1.01, 1.10)* | 0.97 (0.94, 1.00)* | 1.05 (1.01, 1.10)* |
| Pakistani | 1.38 (1.32, 1.45)*** | 1.49 (1.39, 1.59)*** | 1.38 (1.32, 1.45)*** | 1.49 (1.39, 1.59)*** |
| Unknown | 1.02 (0.98, 1.07) | 1.12 (1.04, 1.20)** | 1.02 (0.98, 1.07) | 1.12 (1.04, 1.20)** |
| White And Asian | 1.12 (0.98, 1.28) | 1.13 (0.92, 1.37) | 1.12 (0.98, 1.28) | 1.13 (0.92, 1.37) |
| White And Black African | 1.30 (1.17, 1.43)*** | 1.41 (1.22 1.62)*** | 1.30 (1.17, 1.43)*** | 1.41 (1.22 1.62)*** |
| White And Black Caribbean | 1.38 (1.25, 1.52)*** | 1.41 (1.23 1.61)*** | 1.38 (1.25, 1.52)*** | 1.41 (1.23 1.61)*** |
| **English Proficiency** |  |  |  |  |
| P-English | - | - | - | - |
| P-Unknown | 0.75 (0.73, 0.77)*** | 0.73 (0.70, 0.75)*** | 0.75 (0.73, 0.77)*** | 0.73 (0.70, 0.75)*** |
| P-Other | 0.97 (0.94, 0.99)** | 0.99 (0.96, 1.03) | 0.97 (0.94, 0.99)** | 0.99 (0.96, 1.03) |
| NP-Prev | 1.08 (1.04, 1.13)*** | 1.10 (1.03, 1.17)** | 1.08 (1.04, 1.13)*** | 1.10 (1.03, 1.17)** |
| NP | 1.38 (1.32, 1.45)*** | 1.49 (1.40, 1.59)*** | 1.38 (1.32, 1.45)*** | 1.49 (1.40, 1.59)*** |
| NP-English | 1.38 (1.23, 1.54)*** | 1.58 (1.37, 1.82)*** | 1.38 (1.23, 1.54)*** | 1.58 (1.37, 1.82)*** |
| ^1^*p<0.05; **p<0.01; ***p<0.001 | | | | |

Abbreviations: CI = Confidence Interval; OR = Odds Ratio; IMD = Index of multiple deprivation; P-English = Proficient in English with English as their first language; P-Unknown = Proficient in English, unknown first language; P-Other = Proficient in English, but English is not their first language; NP-Prev = Not proficient in English, captured previously in GP record; NP = Not proficient in English; NP-English *=* English as their first language plus a record indicative of poor English proficiency
